# Lysosome-associated membrane protein 3 misexpression in salivary glands induces a Sjögren’s syndrome-like phenotype in mice

**DOI:** 10.1136/annrheumdis-2020-219649

**Published:** 2021-03-03

**Authors:** Hiroyuki Nakamura, Tsutomu Tanaka, Thomas Pranzatelli, Youngmi Ji, Hongen Yin, Paola Perez, Sandra A Afione, Shyh-Ing Jang, Corrine Goldsmith, Chang Yu Zheng, William D Swaim, Blake M Warner, Noriyuki Hirata, Masayuki Noguchi, Tatsuya Atsumi, John A Chiorini

**Affiliations:** 1 AAV Biology Section, National Institute of Dental and Craniofacial Research, Bethesda, Maryland, USA; 2 Salivary Disorders Unit, National Institute of Dental and Craniofacial Research, Bethesda, Maryland, USA; 3 Division of Cancer Biology, Hokkaido University, Sapporo, Japan; 4 Department of Rheumatology, Endocrinology and Nephrology, Hokkaido University, Sapporo, Japan

**Keywords:** Sjogren's syndrome, autoantibodies, autoimmunity

## Abstract

**Objectives:**

Sjögren’s syndrome (SS) is an autoimmune sialadenitis with unknown aetiology. Although extensive research implicated an abnormal immune response associated with lymphocytes, an initiating event mediated by salivary gland epithelial cell (SGEC) abnormalities causing activation is poorly characterised. Transcriptome studies have suggested alternations in lysosomal function are associated with SS, but a cause and effect linkage has not been established. In this study, we demonstrated that altered lysosome activity in SGECs by expression of lysosome-associated membrane protein 3 (LAMP3) can initiate an autoimmune response with autoantibody production and salivary dysfunction similar to SS.

**Methods:**

Retroductal cannulation of the submandibular salivary glands with an adeno-associated virus serotype 2 vector encoding LAMP3 was used to establish a model system. Pilocarpine-stimulated salivary flow and the presence of autoantibodies were assessed at several time points post-cannulation. Salivary glands from the mice were evaluated using RNAseq and histologically.

**Results:**

Following LAMP3 expression, saliva flow was significantly decreased and serum anti-Ro/SSA and La/SSB antibodies could be detected in the treated mice. Mechanistically, LAMP3 expression increased apoptosis in SGECs and decreased protein expression related to saliva secretion. Analysis of RNAseq data suggested altered lysosomal function in the transduced SGECs, and that the cellular changes can chemoattract immune cells into the salivary glands. Immune cells were activated via toll-like receptors by damage-associated molecular patterns released from LAMP3-expressing SGECs.

**Conclusions:**

These results show a critical role for lysosomal trafficking in the development of SS and establish a causal relationship between LAMP3 misexpression and the development of SS.

**Key messages**

**What is already known about this subject?**

- Lysosome-associated membrane protein 3 (LAMP3) is ectopically expressed in salivary gland epithelial cells (SGECs) of patients with Sjögren’s syndrome (SS).
- LAMP3 overexpression promotes apoptosis and autoantigen release of SGECs in vitro.

**What does this study add?**

- LAMP3 expression in SGECs is directly involved in the development of SS-like phenotype in vivo.

**How might this impact on clinical practice or future developments?**

- The causal relationship between LAMP3 misexpression and SS provides us a novel target for interventions in the treatment of SS.

## Introduction

Sjögren’s syndrome (SS) is an autoimmune disease that primarily affects salivary and lacrimal glands. The disease is characterised by dry mouth and/or eye symptoms, lymphocytic infiltration of the affected glands, and the presence of autoantibodies, such as anti-Ro/SSA and anti-La/SSB antibodies. Although excessive immune activation by type I interferon (IFN) signalling pathway is thought to play an important role in pathogenesis of SS, the aetiology of the disease is unclear.^1^ Genome-wide association studies identified *IRF5* and *STAT4* as susceptibility genes of SS. These genes are involved in IFN production and downstream signalling following stimulation of the IFN receptor.^2–5^ Transcriptome studies of minor salivary gland biopsies and peripheral blood mononuclear cells identified additional IFN signature genes that were increased in patients with SS.^6–9^

Many cellular proteins are reported to be regulated by IFN expression include those associated with lysosomal function. Lysosome-associated membrane protein 3 (LAMP3) is a membrane glycoprotein predominantly localised in lysosomes that is reported to be induced by IFN.^10^ In aggregated microarray studies, increased expression of *LAMP3* is detected in the salivary glands of patients with SS compared with control glands.^11^ However, its causal relationship to the development of SS-associated symptoms is not clear. Functionally, LAMP3 is unique among the LAMP protein members and is specifically expressed in mature dendritic cells and associated with translocation of the major histocompatibility complex class II molecules to the cell surface for antigen presentation.^12^ Confocal immunofluorescence imaging showed that LAMP3 was ectopically expressed in salivary gland epithelial cells (SGECs) as well as in infiltration cells in patients with SS.^11^ Transfection of LAMP3 expression plasmid into SGEC-driven cell lines increased caspase-dependent apoptosis and promoted the release of intracellular SSA and SSB autoantigens via extracellular vesicles.^11^ These in vitro studies suggested LAMP3 expression in SGECs could contribute to the induction of salivary dysfunction and autoantibody production.

The lysosome is a membrane-bound organelle that contains hydrolytic enzymes to degrade many kinds of biomolecules, and has various cellular functions, such as energy metabolism, plasma membrane repair and protein secretion, by ingesting and dissolving cell debris, other damaged organelles or foreign substances that have entered the cell.^13^ Nevertheless, lysosomes have the potential to be harmful to cells if their contained proteases are released to the cytoplasm. The translocation of lysosomal proteases is induced by several cell stresses, and can trigger a cascade of apoptotic pathways.^14^

It is thought that excessive and inappropriate cell death may contribute to the initiation of autoimmunity through the release and ineffective clearance of intracellular antigens.^15^ It has been reported that enhanced apoptosis in SGECs can trigger an SS-like autoimmune disease in mice.^16^ In addition, the relationship between cell death and autoantibody production has been well studied in antineutrophil cytoplasmic antibodies (ANCA)-associated vasculitis. Neutrophil extracellular traps (NETs) are complexes of chromosomal DNA, histones and granule proteins released by neutrophils to bind and kill extracellular pathogens.^17^ The components can be autoantigens when excessive NETs are formed by abnormal neutrophil activation, leading to ANCA production.^18^ Similarly, LAMP3-induced cell death might trigger autoantibody production via inappropriate autoantigen release and activation of immune cells.

To investigate the pathophysiological role of LAMP3 expression in SS, we established a mouse model with LAMP3 overexpressed locally in the submandibular glands following retroductal cannulation with an adeno-associated virus serotype 2 (AAV2) vector encoding LAMP3. The mice develop an SS-like phenotype with progressive salivary hypofunction and anti-Ro/SSA and La/SSB antibody production. Mechanistically, LAMP3 expression increased apoptosis in SGECs as monitored by transferase dUTP nick end labelling (TUNEL) assays and decreased protein expression related to saliva secretion. Activated immune cells were chemoattracted into the salivary glands through the cellular changes in SGECs induced by LAMP3. This study demonstrated a causal link between the development of SS and LAMP3 misexpression, incorrect expression associated with alteration of a phenotype.

## Materials and methods

Detailed explanation of each procedure was described in online supplemental file.

10.1136/annrheumdis-2020-219649.supp1 Supplementary data

Briefly, AAV2 vectors encoding LAMP3 (AAV2-LAMP3) were delivered into both submandibular glands of female 6–8-week-old C57BL/6 mice (10^11^ particles/mouse in 100 µL) by retrograde ductal instillation. The same amount of AAV2 vector encoding green fluorescent protein (AAV2-GFP) was cannulated in control mice. Pilocarpine-stimulated salivary flow and the presence of serum autoantibodies were assessed at several time points post-cannulation. Submandibular glands were evaluated using RNAseq and histologically.

In vitro, LAMP3 expression or empty plasmids were transfected into immortalised acinar and ductal cells derived from normal human salivary glands,^19^ or SGEC-driven cell lines. Protein expression was assessed by western blotting and immunofluorescence. Human monocytic cell line THP-1 cells were treated with supernatant from LAMP3-transfected cells or empty-transfected cells, and transcriptional change was evaluated using quantitative real-time reverse transcription PCR (qRT-PCR).

## Results

### LAMP3 is increased in expression with disease onset in non-obese diabetic mice

**Figure 1:**
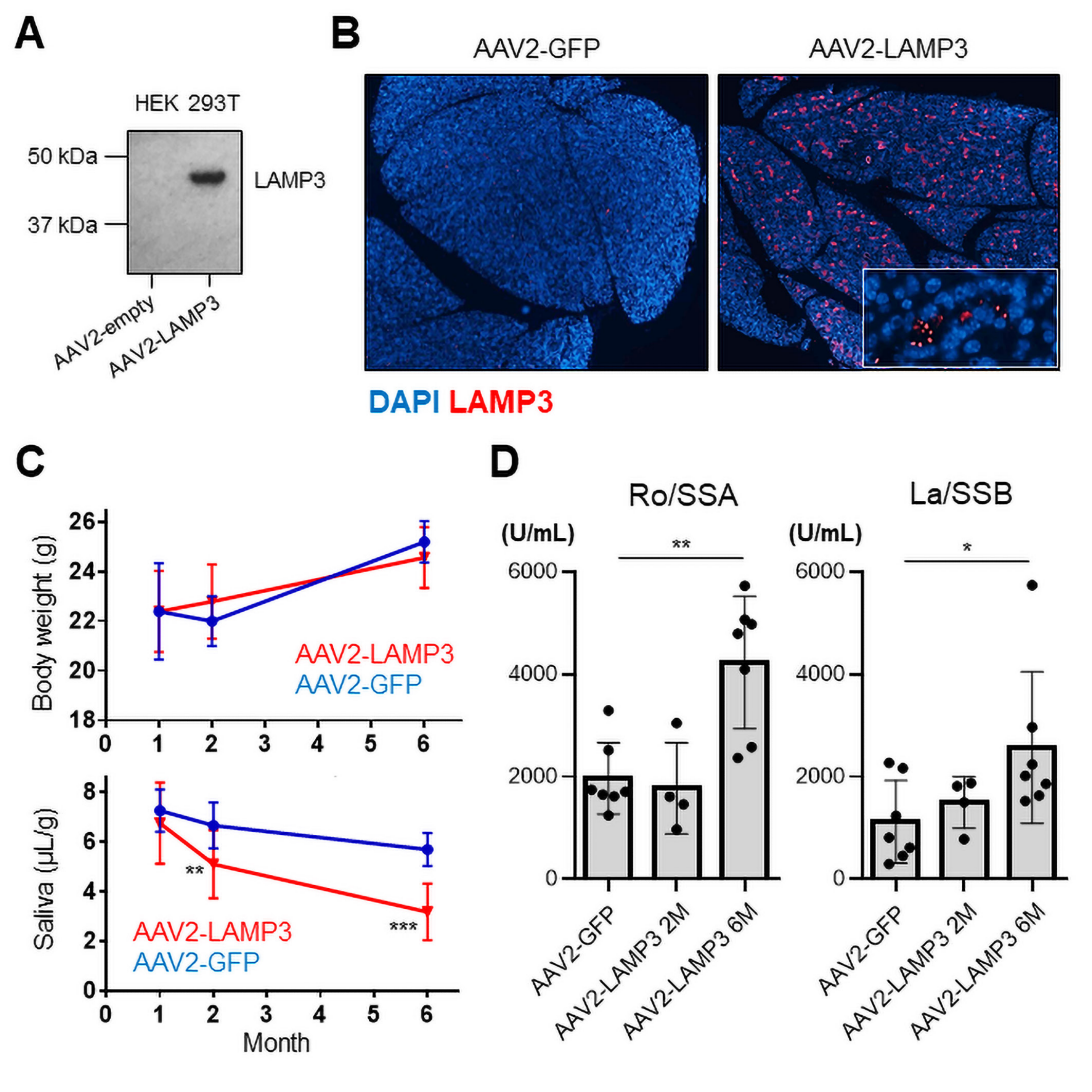
Ectopic expression of LAMP3 in salivary gland induces a Sjögren’s syndrome-like salivary dysfunction and autoantibody production in mice. (A) Immunoblotting of HEK 293T cell lysate 48 hours after transfection with AAV2-LAMP3 or AAV2-empty plasmids. (B) Immunofluorescent images (4× magnification) in murine submandibular glands 2 months after transduction with AAV2-LAMP3 or AAV2-GFP. (C) Body weight and pilocarpine-stimulated salivary flow per body weight in 20 min in AAV2-LAMP3 treated mice (N=20) and control AAV2-GFP treated mice (N=10). (D) Serum anti-Ro/SS-A and anti-La/SS-B antibodies were measured in AAV2-GFP treated mice (N=7), 2 months after transduction with AAV2-LAMP3 (N=4) and 6 months after AAV2-LAMP3 (N=7). Values are shown as the mean±SD. *p<0.05, **p<0.01, ***p<0.001, t-test with Bonferroni’s correction. LAMP3, lysosome-associated membrane protein 3.

The non-obese diabetic (NOD) mouse is a well characterised model of spontaneous onset of an SS-like phenotype in female mice at approximately 16 weeks of age independent of the occurrence of loss of glycaemic control.^20 21^ Comparison of transcriptome data between patients and this model has shown conservation of phenotype associated patterns of gene expression.^22^ Investigation of LAMP3 expression revealed similar levels of LAMP3 protein in salivary glands from 8-week-old NOD mice compared with C57BL/6 mice. By 20 weeks, LAMP3 expression had increased in the NOD mice compared with the controls (online supplemental S-Figure 1), suggesting as with the patients, LAMP3 expression is associated with disease progression in this mouse model.

### AAV2-LAMP3 induces hyposalivation and autoantibodies in mice

**Figure 2:**
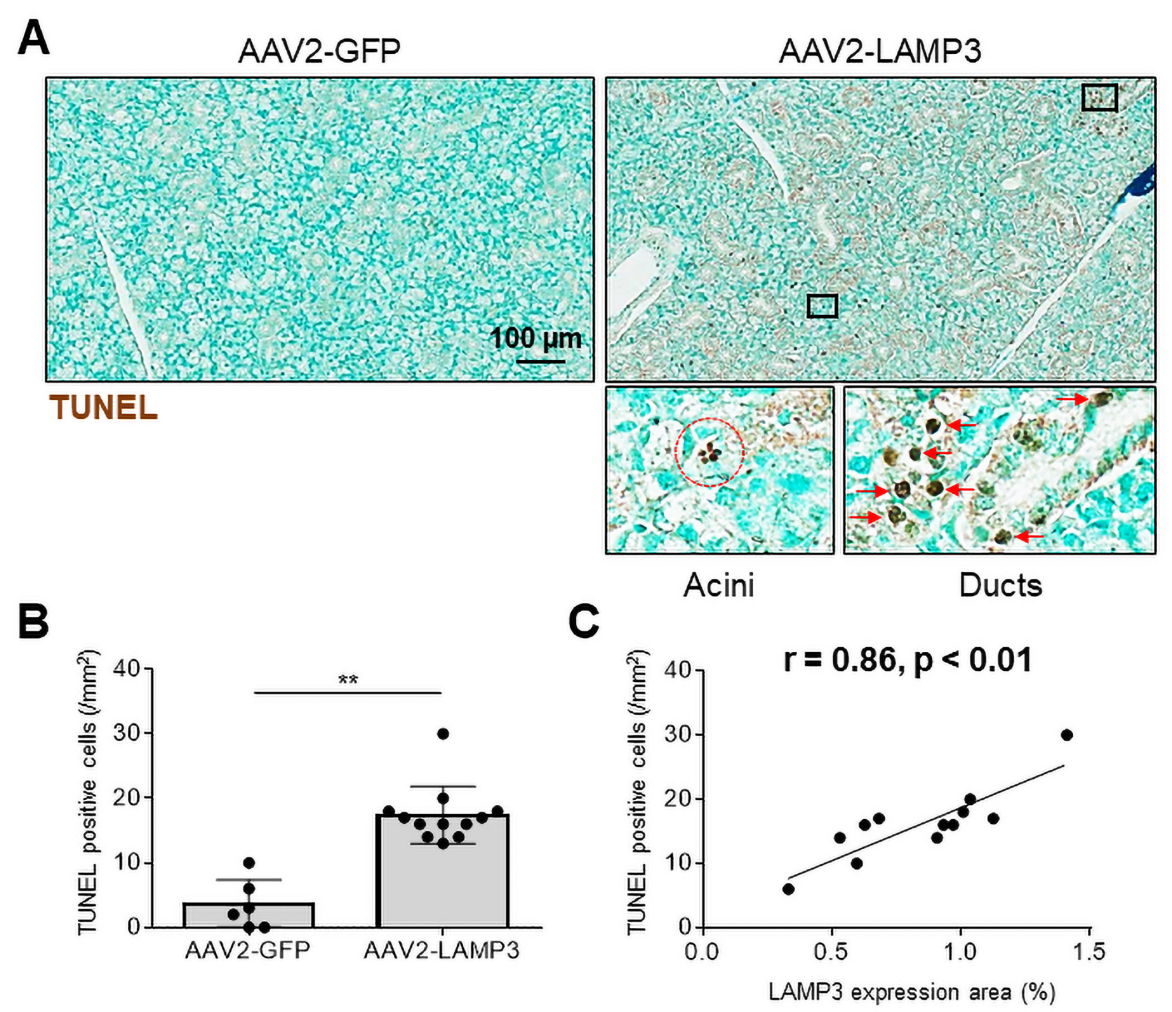
Transduction with AAV2-LAMP3 vector induces apoptosis in murine salivary gland epithelial cells. (A) Representative submandibular gland specimens with terminal deoxynucleotidyl transferase dUTP nick end labelling (TUNEL) from mice 2 months post-cannulation with AAV2-LAMP3 (N=6) or AAV2-GFP (N=12). Brown staining indicates apoptotic cells. (B) The number of TUNEL positive (apoptotic) cells were quantified. Values are shown as the mean±SD. **p<0.01, t-test. (C) Correlation between the number of TUNEL positive (apoptotic) cells and LAMP3 expression area in submandibular gland specimens. Dots shows the result from each murine specimen. LAMP3, lysosome-associated membrane protein 3.

To establish a causal relationship between LAMP3 expression and the development of SS-associated pathology, LAMP3 expression was initiated in the salivary glands of healthy C57BL/6 mice by retroductal cannulation of the submandibular glands using AAV2 vectors to facilitate gene expression. AAV2 vectors were chosen because of their ability to direct long-term expression following localised delivery to the salivary glands of mice, with minimal host response to the vector.^22^ Initial experiments demonstrated that western blotting of HEK 293T cells transfected with AAV2-LAMP3 plasmids showed expression of a 44 kDa protein corresponding to the reported molecular weight of LAMP3^12^ (figure 1A). Confocal immunofluorescence imaging of AAV2-LAMP3 vector cannulated mice showed extensive and sustained expression of LAMP3 in the murine submandibular glands 2 months post-cannulation, which appeared as a puncta pattern in the salivary tissue similar to previously reports.^23^ Close inspection revealed that AAV2-LAMP3 expressed protein was predominantly localised to salivary epithelial cells, consistent with the tropism of AAV2 in the submandibular salivary gland^24 25^ (figure 1B). GFP expression was confirmed in the submandibular glands of AAV2-GFP cannulated mice (online supplemental S-Figure 2).

The effects of LAMP3 were assessed overtime between 1 and 6 months post-cannulation. Expression of LAMP3 in the salivary glands did not appear to have an overt effect on feeding by the mice as there was no significant differences in body weight between AAV2-LAMP3 treated mice (LAMP3 mice) and control mice (figure 1C). Pilocarpine-stimulated salivary flow in LAMP3 mice and controls was tested at several time points following cannulation. This analysis over time showed that saliva flow did not change significantly 1-month post-cannulation (6.8 µL/g vs 7.3 µL/g, p=0.51), but was markedly decreased by 2 months post-cannulation (5.1 µL/g vs 6.7 µL/g, p<0.01) and continued to decrease 6 months post-cannulation (3.2 µL/g vs 5.7 µL/g, p<0.001) in LAMP3 mice compared with controls (figure 1C). Anti-Ro/SSA and anti-La/SSB antibodies were detected in sera from LAMP3 mice 6 months post-cannulation (figure 1D), consistent with that LAMP3 expression was associated with serum anti-Ro/SSA and/or anti-La/SSB in patients with SS, and that in vitro LAMP3 expression promoted the accumulation and release of SSA and SSB autoantigens.^11^ These findings suggest that LAMP3 expression in vivo can stimulate an SS-like phenotype in mice.

### LAMP3 induces apoptosis of SGECs in mice

Previous work reported that transfection of LAMP3 expression plasmids into SGEC derived cells grown in culture induced apoptosis.^11^ To investigate if LAMP3 expression causes apoptosis of SGECs in vivo, salivary gland tissue from LAMP3 and control mice were assayed by TUNEL staining and imaged using light microscopy. Apoptotic TUNEL positive cells were abundant in acini and ducts of the salivary glands of the LAMP3 mice (figure 2A). The number of the apoptotic cells were significantly increased in the LAMP3 mice compared with control mice (15.1/mm^2^ vs 4.2/mm^2^, p<0.01, figure 2B). Furthermore, the number of apoptotic cells had a significant positive correlation with LAMP3 expression in the murine submandibular specimens (r=0.86, p<0.01, figure 2C). These findings demonstrate that LAMP3 expression increased apoptosis in SGECs in vivo.

### Lysosomal and immune pathways were activated in LAMP3 mice

**Figure 3:**
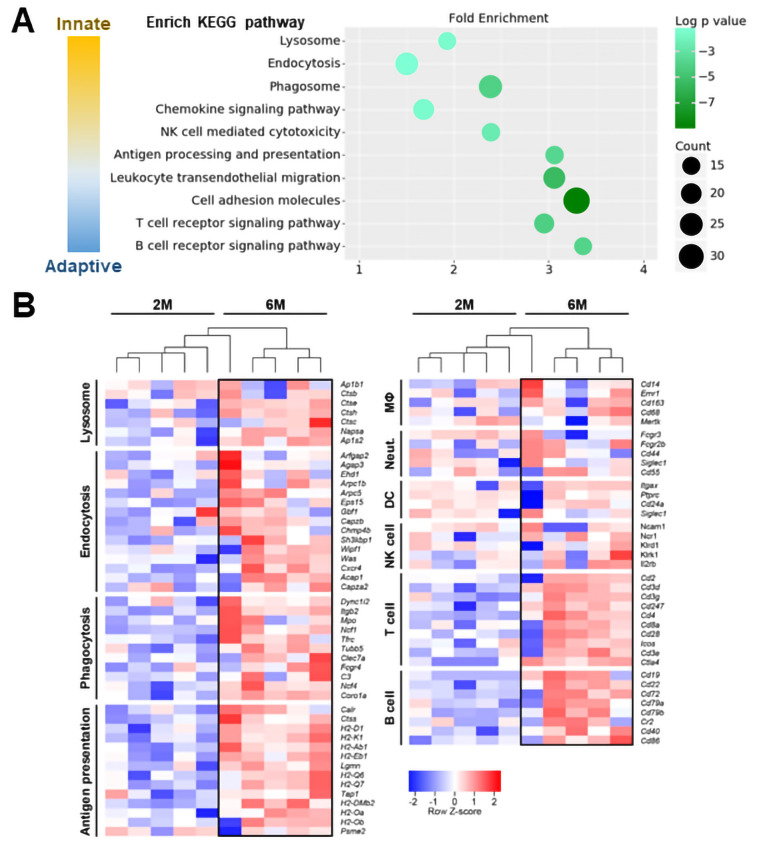
Lysosomal and immune activation is induced in submandibular glands of AAV2-LAMP3 mice 6 months after transduction. (A) The scatter plot lists significantly upregulated KEGG pathways in AAV2-LAMP3 mice 6 months post-cannulation (N=5) compared with 2 months post-cannulation (N=5). (B) The heat map showing the relative expression of the signature genes for the indicated functions or cell types in submandibular glands from AAV2-LAMP3 mice 2 months (2M) and 6 months (6M) post-cannulation. DC, dendritic cell; MΦ, macrophage; NK, natural killer; Neut., neutrophil.

To clarify the molecular mechanism associated with the development of an SS-like phenotype in the LAMP3 mice, RNA was collected from the submandibular glands of AAV2-LAMP3 cannulated mice at 2 months and 6 months post-cannulation and used for RNAseq and bioinformatics analysis. A total of 1063 differentially expressed genes were identified between the two groups (701 genes were upregulated, and 362 downregulated at 6 months). Analysis using DAVID Bioinformatics Resources 6.8 identified the differentially expressed genes were enriched in lysosome, endocytosis, phagocytosis, chemokine signalling, natural killer cell mediated cytotoxicity, antigen processing/presentation, leucocyte migration, adhesion and T/B-cell receptor signalling pathways. These enrichment pathways were related to innate and adaptive immunity (figure 3A).

At 6 months post-cannulation of AAV2-LAMP3, genes associated with lysosomal membrane proteins, adaptor proteins and proteases were upregulated compared with the 2 months time point, suggesting that LAMP3 alters lysosomal function in the transduced SGECs. One interpretation of the RNAseq data is that apoptosis observed in the SGECs lead to macrophage activation to remove the apoptotic cells, supported by upregulation of the genes associated with phagocytosis-promoting receptors and antigen presentation in LAMP3 mice 6 months post-cannulation. The interaction between apoptotic SGECs and macrophages is considered to be critical to initiate autoimmunity. Subsequently, upregulated chemokine signalling, cell migration and adhesion molecules would attract lymphocyte in salivary gland tissues. Antigen presenting cells (APCs), T-cells and B-cell related genes were increased in the submandibular glands of LAMP3 mice at 6 months post-cannulation, reflecting cellular infiltration (figure 3B). Additional analysis of the RNAseq data of the immune infiltrate within salivary glands of LAMP3 mice suggested that Th1 and Th17 were the dominant T-cell subtypes as some of their signature genes were significantly upregulated including *Tbx-21, Jak1, Stat3/4* and *Ccr5/6* (online supplemental S-Figure 3). Taken together these data suggest LAMP3 expression in SGECs activate immune cells in a time dependent manner.

**Figure 4:**
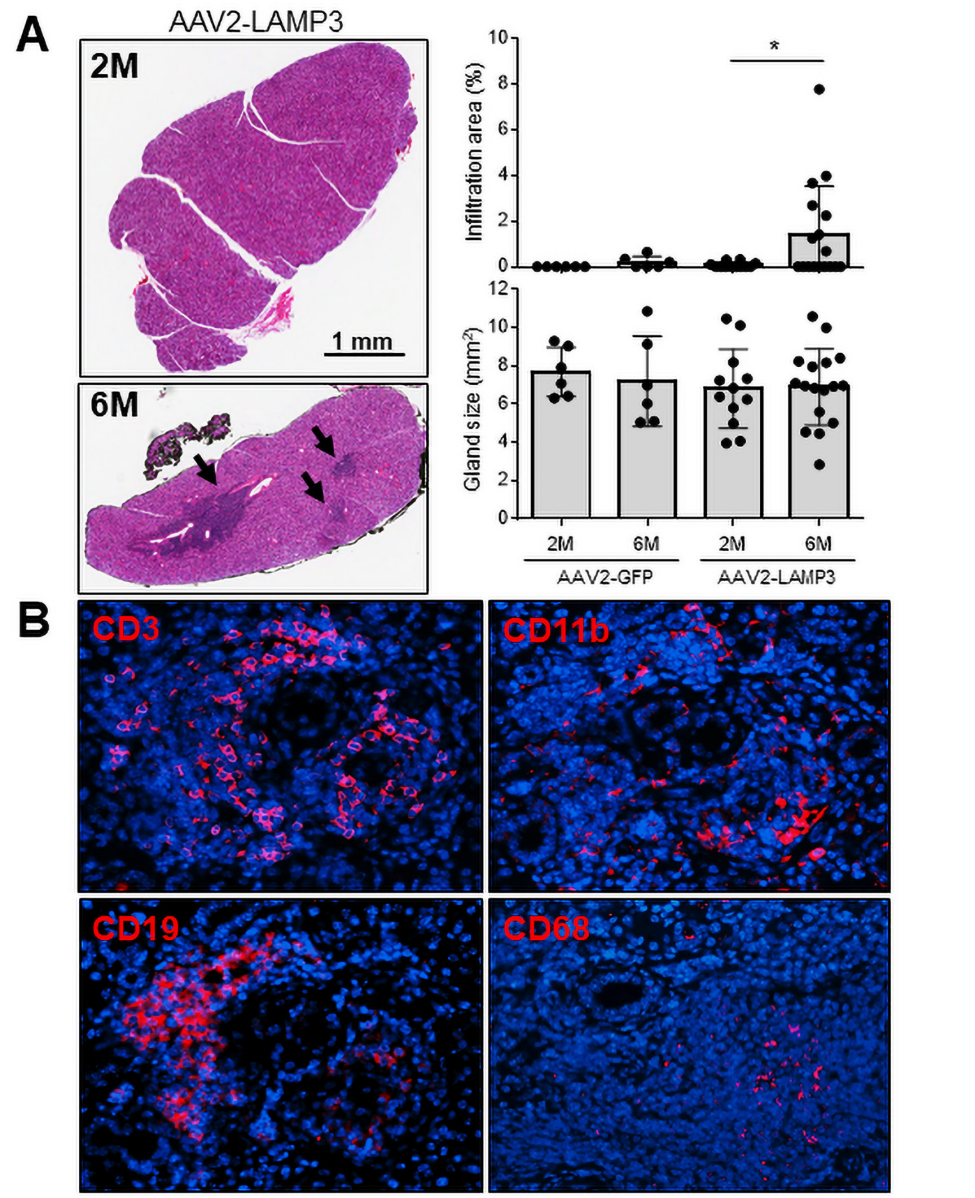
Immune cell infiltration increases in submandibular glands of AAV2-LAMP3 mice 6 months after transduction. (A) Representative H&E staining of submandibular glands from AAV2-LAMP3 mice 2 months (2M) and 6 months (6M) post-cannulation. Black arrows indicate lymphatic infiltration. Salivary gland size and infiltration area were quantified in AAV2-GFP treated mice 2M (N=6) and 6M (N=6) post-cannulation and AAV2-LAMP3 treated mice 2M (N=10) and 6M (N=17) post-cannulation. Values are shown as the mean±SD. *p<0.05, t-test with Welch’s correction. (B) Representative immunofluorescent images (40× magnification) in murine submandibular glands 6M post-cannulation with AAV2-LAMP3. LAMP3, lysosome-associated membrane protein 3.

**Figure 5:**
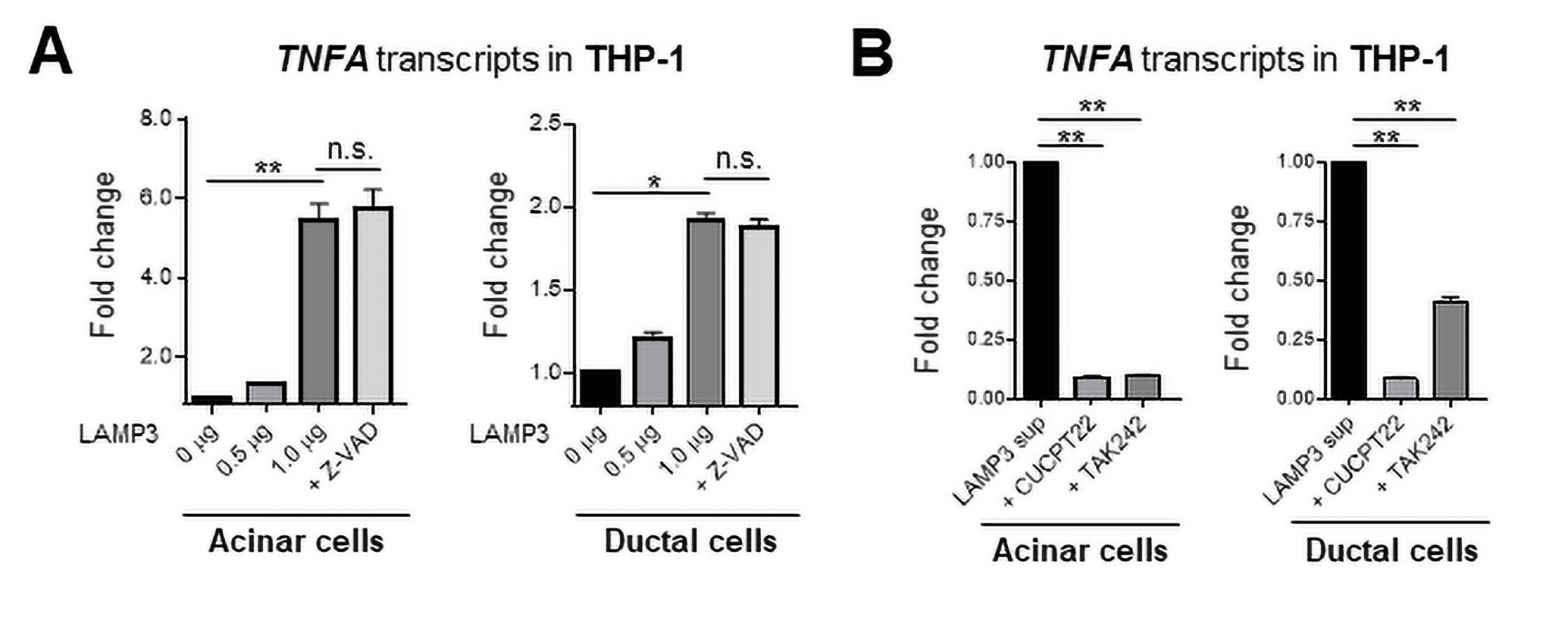
Culture supernatant from LAMP3-expressing epithelial cells activates THP-1 cells. (A) Acinar and ductal cells were transfected with LAMP3 expression or empty plasmids ± Z-VAD (20 µM). (B) THP-1 cells were stimulated with the culture supernatant collected from LAMP3-transfected cells 96 hours after transfection ± CUCPT22 (20 µM) or TAK242 (40 µM). Transcript changes in THP-1 cells were evaluated 20 hours after stimulation. Values are shown as the mean±SEM from independent three experiments. *p<0.05, **p<0.01, t-test with Bonferroni’s correction. LAMP3, lysosome-associated membrane protein 3.

In agreement with the increase immune activation suggested by the analysis of the RNAseq data, H&E staining of slides identified clusters of lymphocytic infiltration in submandibular gland specimens from LAMP3 mice 6 months post-cannulation (figure 4A). The lymphocytic infiltration area was not significantly correlated with serum autoantibodies or saliva flow (online supplemental S-Figure 4). Murine submandibular glands were visualised by immunofluorescences using CD3, CD19, CD11b and CD68 antibodies, confirming the infiltration of T-cells, B-cells and APCs including CD68^+^ macrophages in the tissues (figure 4B). These positive signals were abundantly found in the LAMP3 mice 6 months post-cannulation compared with those 2 months post-cannulation (online supplemental S-Figure 5). T-cells were dominant (58%) among the infiltration cells, followed by B-cells (29%) and APCs (13%).

**Figure 6:**
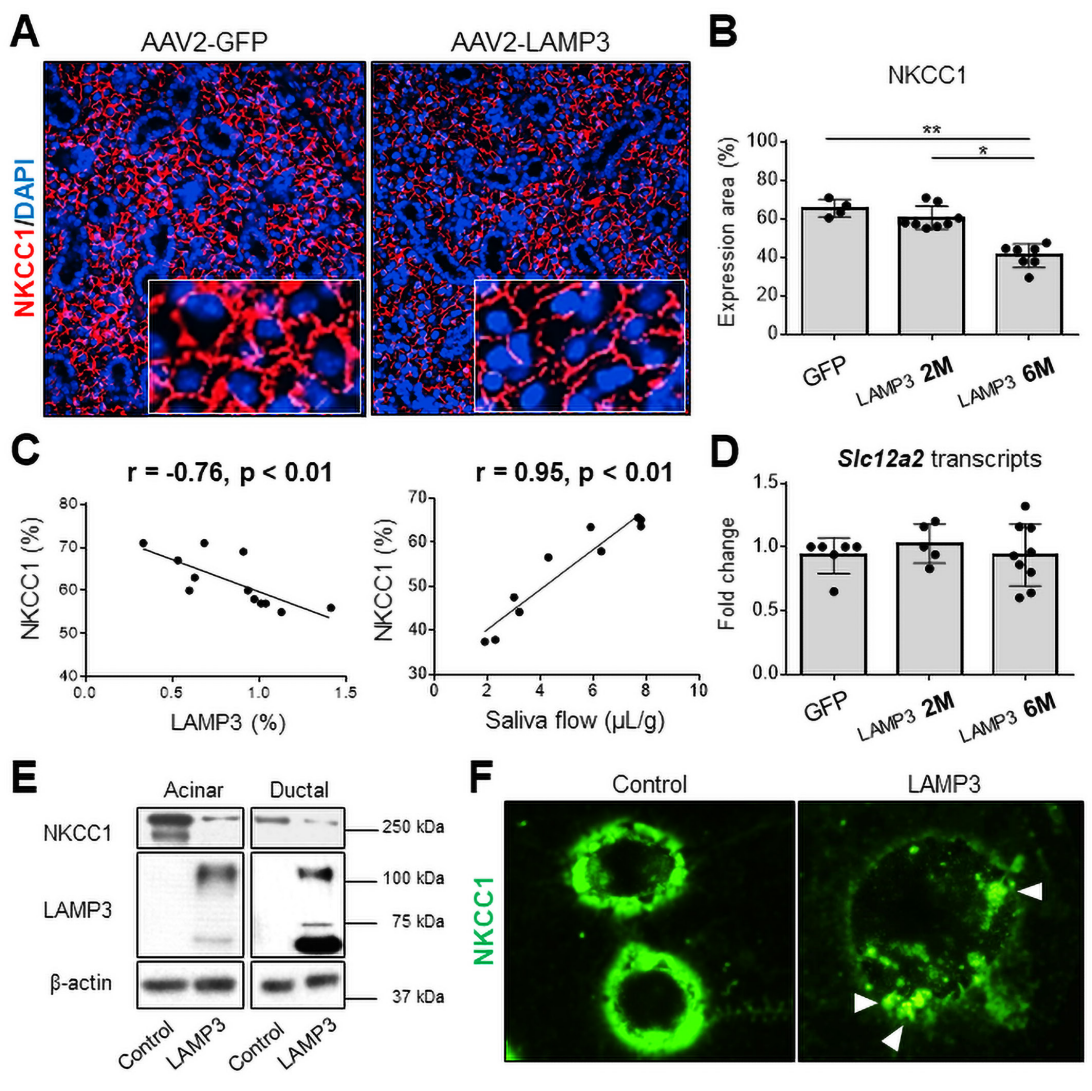
LAMP3 expression decreases NKCC1 expression in salivary gland epithelial cells. (A) Immunofluorescence images in murine submandibular glands (40× magnification) from mice treated with AAV2-LAMP3 or AAV2-GFP. (B) Expression area of NKCC1 was quantified in AAV2-GFP treated mice (N=4) and AAV2-LAMP3 treated mice 2 months (2M) post-cannulation (N=8) and 6 months (6M) post-cannulation (N=7). (C) Correlation between the NKCC1 and LAMP3 expression in submandibular gland specimens, or saliva flow. (D) Transcript change of *Scl12a2* gene in submandibular gland tissues from AAV2-GFP treated mice (N=6) and AAV2-LAMP3 treated mice 2M (N=5) and 6M (N=9) post-cannulation. (E) Western blotting with indicated antibodies using acinar or ductal cell lysate 72 hours post-transfection with LAMP3 or empty plasmids. (F) Immunofluorescence images (40× magnification) of A253 cells 48 hours after transfection with LAMP3 or empty plasmids. Representative images from three independent experiments. Values are shown as the mean±SD. *p<0.05, **p<0.01, t-test with Bonferroni’s correction. LAMP3, lysosome-associated membrane protein 3; NKCC1, Na-K-Cl cotransporter-1.

Taken together, the bioinformatics analysis supports a hypothesis that LAMP3 expression in SGECs can be stimulatory to monocytes/macrophages as an initiator of autoimmunity. To test the hypothesis, THP-1 cells were treated in vitro with supernatant from LAMP3-transfected salivary gland acinar cells or ductal cells and stimulation was monitored by qRT-PCR for changes in cytokine production. Treatment of THP-1 cells with the supernatant stimulated a significant increase in the transcription of TNFα compared with control treated THP-1 cells. The increased expression of TNFα in THP-1 cells was independent of apoptosis in acinar or ductal cells as treatment with the pan-caspase inhibitor Z-VAD did not inhibit the increase in TNFα expression (figure 5A). In contrast, the increase in TNFα expression was inhibited in THP-1 cells by treatment with toll-like receptor (TLR) 1/2 antagonist (CUCPT22) or TLR 4 antagonist (TAK242) (figure 5B, online supplemental S-Figure 6). These TLRs recognise damage-associated molecular patterns released from the cells. Previous in vitro study showed that LAMP3 expression promoted the release of SSA and SSB autoantigens from SGEC-driven cell lines,^11^ which could be recognised and be processed by activated macrophages in salivary glands, leading to antibody production against the autoantigens.

### LAMP3 degrades functional proteins and contributes to salivary hypofunction in mice

**Figure 7:**
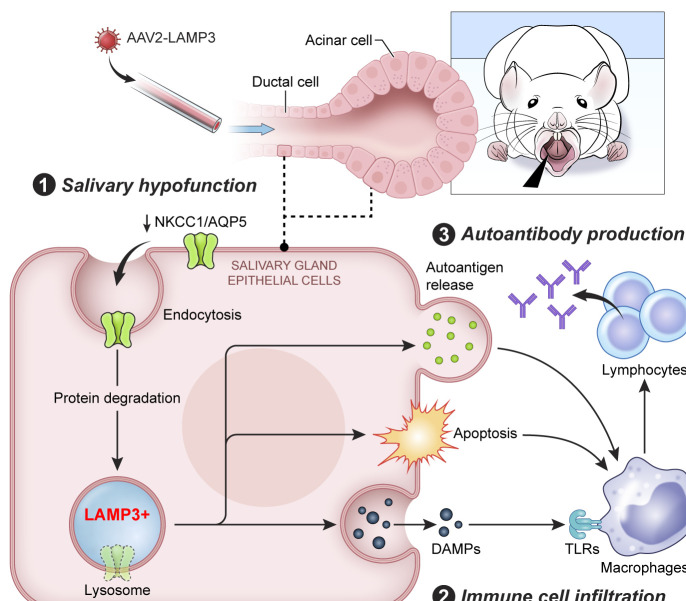
Graphical abstract. Lysosome-associated membrane protein 3 (LAMP3) misexpression in salivary gland epithelial cells (SGECs) followed by retroductal cannulation with an AAV2-LAMP3 vector induces salivary dysfunction, immune cell infiltration and autoantibody production in mice. Mechanistically, LAMP3 misexpression altered lysosomal function in the transduced SGECs, increased apoptosis, promoted the release of intracellular autoantigens and decreased protein expression related to saliva secretion, such as Na-K-Cl cotransporter-1 (NKCC1) and aquaporin 5 (AQP5). Immune cells were activated via toll-like receptors (TLRs) by damage-associated molecular patterns (DAMPs) released from SGECs.

Despite the increase of apoptosis in the salivary glands of LAMP3 mice, overall atrophy or destruction of the gland structure was not seen at either 2 or 6 months post-cannulation (figure 4A). There was also no significant difference in gene expression associated with the progressive decrease in saliva secretion observed between 2 and 6 months in the RNAseq data from the LAMP3 treated mice (online supplemental S-Figure 7). Further analysis of the RNAseq data suggested that LAMP3 might promote the degradation of proteins at post-transcriptional level through altered lysosomal and endocytic activities (figure 3). To further investigate the salivary hypofunction associated with salivary gland targeted LAMP3 expression, slides from the mice were compared for expression of membrane proteins related to salivary gland function. Na-K-Cl cotransporter-1 (NKCC1) and aquaporin 5 (AQP5) are common markers of SGECs and their expression are closely linked to changes in salivary gland activity.^26 27^ Immunofluorescent imaging showed decreased expression of NKCC1 (figure 6A) and AQP5 (online supplemental S-Figure 8A) proteins in submandibular glands of LAMP3 mice compared with control mice cannulated with a vector encoding GFP. Expression of NKCC1 (figure 6B) and AQP5 (online supplemental S-Figure 8B) was significantly decreased in LAMP3 mice 6 months after transduction compared with GFP control mice and 2 months LAMP3 mice. Overall the decrease in expression of NKCC1 and AQP5 as measured by area was negatively correlated with LAMP3 expression and was positively correlated with saliva flow (figure 6C and online supplemental S-Figure 8C, respectively). In contrast, transcript levels of *Scl12a2* (encoding NKCC1, figure 6D) and *Aqp5* (online supplemental S-Figure 8D) genes measured by qRT-PCR in the submandibular gland tissues were similar in GFP control mice, 2 months and 6 months LAMP3 mice. Western blotting of cell lysate from LAMP3 transfected cells showed decrease expression of NKCC1 (figure 6E) and AQP5 (online supplemental S-Figure 8E) when normalised to β-actin compared with control transfected cells. Finally, immunocytochemistry staining showed decreased fluorescence intensity of NKCC1 protein on the cell membrane and formation of endocytic vesicles including NKCC1 (white arrow heads) in LAMP3-transfected cells (figure 6F). Taken together these data suggest a physiological mechanism involved in the decreased salivary gland secretion associated with LAMP3 expression, which is caused by increased membrane turnover and endocytic lysosomal activity of the SGECs resulting in degradation of membrane proteins critical for saliva secretion.

## Discussion

Increased IFN signalling is well established as a component of the pathogenesis associated with SS, but little is known regarding the effect of IFN on epithelial cells and its causal association with salivary gland hypofunction and the induction of autoimmunity. We recently identified by aggregated microarray analysis that LAMP3, an IFN inducible protein, is upregulated in patients with SS. Further analysis identified an association between LAMP3 expression levels and autoantibody positivity in patients. In vitro studies suggested that LAMP3 could induce apoptosis and the accumulation and release of SS-associated autoantigens via extracellular vesicles.^11^ In this study, we have extended these findings and demonstrated that overexpression of LAMP3 in vivo can induce an SS-like phenotype in mice. Analysis of RNAseq data suggested that LAMP3 expression resulted in altered lysosome activity and immune cell activation. In vivo LAMP3 expression induced apoptosis of SGECs, immune infiltration in the glands and degradation of membrane proteins associated with saliva secretion. In vitro studies suggest that LAMP3 expression in SGECs can trigger activation of monocytes/macrophages (figure 7).

A number of mouse models of SS have been reported.^28^ Intraperitoneal injections of SSA/Ro antigen induce immune activation and an SS-like phenotype in mice.^29 30^ Other models show that expression of nterleukin (IL)-12,^31^ IL-14^32^ or IL-17A^33 34^ can induce an SS-like disease profile in transgenic mice through T-cell and B-cell activation and differentiation. T-cell-targeted deletion of stromal interaction molecule 1 and 2 in T-cells stimulated spontaneous and severe SS-like autoimmune disease in mice.^35^ Together, these models support an association between the development of SS and dysregulation of the immune system. Our data from the LAMP3 overexpression mouse suggests that the lysosomal changes in SGECs could be responsible for the initiation of autoimmunity. Another model system is NOD mice that spontaneously develop SS-like autoimmune exocrinopathy as well as type I diabetes.^20^ We found that LAMP3 expression was also increased in NOD mice with age. Lysosomal dysfunction is involved in various diseases including cancer,^36^ Alzheimer’s disease^37^ and Parkinson’s disease.^38^ Our data suggest that lysosomal proteins and function also has an important role in SS.

It is still unclear the mechanism that results in the increased LAMP3 expression in the SGECs of patients with SS. Considering that LAMP3 is an IFN-inducible gene, environmental factors like viral infection^39 40^ and genetic susceptibility, such as *IRF5* and *STAT4* polymorphisms,^2–5^ might trigger LAMP3 misexpression. These considerations suggest that inhibition of the IFN pathways might be a possible intervention to prevent the increased LAMP3 expression in the salivary glands. In support of this intervention, recent work demonstrated that JAK inhibitors can ameliorated SS-like manifestations in NOD mice through downregulating IFN pathways.^41^ Future studies are needed to clarify the pathological connection between IFN signalling and LAMP3 misexpression in patients with SS.

In conclusion, this study shows that LAMP3 expression in SGECs can induce an SS-like phenotype. The development of disease in this mouse model has distinct phases with the induction of apoptosis preceding salivary hypofunction, followed by progressive levels of immune activation, ultimately leading to the development of autoimmunity. Further investigations are needed to understand the context of the release of autoantigens from the cell and if additional cellular or lysosomal components are necessary for the induction of autoimmunity. Taken together, the present results demonstrate a critical role for salivary epithelial lysosomes in the development of SS and provide a new model for studies of targeted therapeutic interventions in SS.

## Data Availability

Data are available upon reasonable request. All data relevant to the study are included in the article or uploaded as supplementary information. Data are available from the corresponding author upon request.
