# Supplementary material for: Lysosome-associated membrane protein 3 misexpression in salivary glands induces a Sjögren’s syndrome-like phenotype in mice
Source: Ann Rheum Dis. 2021 Mar 3;80(8):1031–9. doi: 10.1136/annrheumdis-2020-219649 (PMC8292598; doi:10.1136/annrheumdis-2020-219649)
Supplement: Supplementary data [file annrheumdis-2020-219649supp001.pdf]

Lysosome-associated membrane protein 3 misexpression in salivary glands induces a Sjögren's syndrome-like phenotype in mice

Hiroyuki Nakamura[1]*, Tsutomu Tanaka[1]*, Thomas J.F. Pranzatelli[1], Youngmi Ji[1], Hongen Yin[1], Paola Perez[1], Sandra A Afione[1], Shyh-Ing Jang[1], Corrine Goldsmith[1], Changyu Zheng[1], William D. Swaim[1], Blake M. Warner[1], Noriyuki Hirata[2], Masayuki Noguchi[2], Tatsuya Atsumi[3] and John A. Chiorini[1]

1) Adeno-Associated Virus Biology Section, National Institute of Dental and Craniofacial Research, National Institutes of Health, Bethesda, MD, USA.

2) Division of Cancer Biology, Institute for Genetic Medicine, Hokkaido University, Sapporo, Japan

3) Department of Rheumatology, Endocrinology and Nephrology, Faculty of Medicine, Hokkaido University, Sapporo, Japan

Correspondence to: John A. Chiorini.

AAV Biology Section, NIH 10 Center Dr. Bethesda MD 20892.

Phone: 301-496-4279 (Fax 301-402-1228)

Email: jchiorini@dir.nidcr.nih.gov

Nakamura H, *et al. Ann Rheum Dis* 2021; 80:1031–1039. doi: 10.1136/annrheumdis-2020-219649

SUPPEMENTAL MATERIALS AND METHODS

Cells

Human cell line HEK 293T cells were cultured in DMEM (Thermo Fisher Scientific, USA) supplemented with 10% fetal bovine serum (FBS). Immortalized acinar cells and ductal cells derived from normal human salivary glands[1], were donated by Professor M. Azuma, and were cultured in keratinocyte serum-free medium and in keratinocyte grown medium-2 (Lonza, Switzerland), respectively. Human leukemia monocytic cell line THP-1 cells were purchased from American Type Culture Collection (ATCC, USA), and were grown in RPMI-1640 culture medium (Thermo Fisher Scientific) supplemented with 10% FBS, 2mM L-glutamine and 0.05 mM 2-mercaptoethanol. Human cell line HSG cells provided by Dr. Indu Ambudkar were cultured in DMEM (Thermo Fisher Scientific) supplemented with 10% FBS. Human salivary gland cell line A253 cells were purchased from ATCC, and were cultured in McCoy'5A Medium (Thermo Fisher Scientific) supplemented with 10% FBS. All cells were incubated at 37°C with humidity in 5% CO2.

Expression plasmids of pME18S-empty and pME18S-LAMP3 were prepared by cloning the LAMP3 open reading frame into pME18S expression vector containing a Kozak consensus sequence[2]. The plasmids were purified using Endofree plasmid maxi kit (QIAGEN, USA). Acinar cells and ductal cells were transfected with a total amount of the 1.0 µg plasmids per $1{\times}10^6$ cells using Amaxa Nucleofector (Lonza). HSG and A253 cells were transfected with a total amount of the 3.0 µg plasmids per $1{\times}10^6$ cells using Lipofectamine 3000 (Thermo Fisher Scientific).

Adeno-associated virus (AAV) Vector Preparation

HEK 293T cells were cotransfected with the AAV serotype 2 (AAV2) Rep-Cap plasmid, adenoviral helper plasmid and green fluorescent protein (GFP) or LAMP3 expression plasmid by calcium phosphate precipitation to establish AAV2 virus encoding LAMP3 (AAV2-LAMP3) and control (AAV2-GFP). The cell lysate was harvested 48 hours after transfection and were adjusted to a refractive index of 1.372 by addition of cesium chloride and centrifuged at 38,000 g for 65 hours at 20°C. Equilibrium density gradients were fractionated and fractions with a refractive index of 1.369 to 1.375 were collected, and were stored at -80°C. The number of AAV2 particles was estimated by quantitative-polymerase chain reaction, and the vector was dialyzed for 3 hours against saline to adjust the concentrations of 1×10^{12} particles/mL.

Animals

Female 6-8-week-old C57BL/6 mice were obtained from Charles River. Animals were housed in a pathogen-free facility. All procedures involving live animals were performed in an accredited vivarium according to institutional guidelines and standard operating procedures and were following the NIH Guide for the Care and Use of Laboratory Animals. AAV2-GFP or AAV2-LAMP3 virus vectors were delivered into the both submandibular glands (10^{11} particles/mouse in 100 µl) by retrograde ductal instillation through a thin cannula under intramuscular anesthesia with ketamine and xylazine. Body weight and pilocarpine-stimulated salivary flow rate in 20 minutes were determined at several time points post-cannulation. Blood and whole salivary gland tissues were collected at the end of the study. Serum was separated by centrifugation, and then was stored at -80°C. Half of the tissue was fixed with 10% neutral buffered formalin (NBF), embedded in paraffin, and sectioned at 5 µm. The other half of tissue was stored in RNAlater (Thermo Fisher Scientific) at -80°C.

Determination of anti-Ro/SSA and anti-La/SSB antibodies in sera

Anti-Ro/SSA and anti-La/SSB antibodies were tested by solid-phase enzyme-linked immunosorbent assays, according to the manufacturer's instructions (Alpha Diagnostic International, USA). Briefly, serum samples were added to microtiter plates coated with purified Ro/SSA or La/SSB, followed by hydrogen peroxidase (HRP) anti-mouse IgG antibodies. Optical density (OD) at 450 nm was measured using FLUOstar Omega (BMG Labtech, USA) after chromogenic substrate was added.

TUNEL assay

TUNEL was assayed according to the manufacturer's instructions (Abcam, USA). Briefly, deparaffinized and rehydrated specimens were processed with proteinase K and 3% H_20_2, and then were incubated with biotin-labeled terminal deoxynucleotidyl transferase at 37°C for 90 minutes. After washing, HRP conjugate was added, followed by reaction with diaminobenzidine solution and counterstaining with methyl green.

Immunofluorescence staining

Cells in 8 well chamber slides were fixed with 10% NBF, and then were washed with phosphate-buffered saline (PBS). Formalin-fixed paraffin embedded sections were deparaffinized, rehydrated, and subjected to citric acid microwave antigen retrieval. Slides

Nakamura H, *et al. Ann Rheum Dis* 2021; 80:1031–1039. doi: 10.1136/annrheumdis-2020-219649

were blocked with 2% bovine serum albumin (Sigma Aldrich, USA) in PBS for 1 hour at 25°C in a humidified chamber then incubated at 4°C overnight with primary antibodies as listed, 1 μg/mL of CD3, CD19, CD11b, CD68 and GFP (Abcam); 10 μg/mL of LAMP3 (Proteintech, USA), AQP5 (Alomone Labs, Israel) and NKCC1 (Cell Signaling Technology, USA). Slides were then washed in 5 changes of PBS for 5 minutes each, and then incubated with 10 μg/mL AlexaFluor 488 or 594 donkey anti-rabbit IgG (Jackson ImmunoResearch, USE) secondary antibody for 1 hour at 25°C in the dark, followed by washing in 5 changes of PBS for 5 minutes each, and counterstaining with DAPI mounting medium. All images were acquired by Nikon fluorescent microscope. Quantification of expression were performed by ImageJ software (public domain-National Institutes of Health, USA).

RNAseq and bioinformatics analysis

Total RNA was extracted from murine submandibular tissues using the RNeasy Mini Kit (QIAGEN, USA), and was treated with TURBO DNase (Thermo Fisher Scientific). RNA quality and concentration were determined of a Fragment Analyzer instrument (Agilent, USA). Total RNA was reverse transcribed by Superscript IV (Invitrogen, USA) using template switching oligo and oligo dT primers followed by amplification of the second strand cDNA with LongAmp Taq polymerase (New England Biolabs, USA). Libraries were prepared using the Nextera XT method (Illumina, USA), individually barcoded, pooled to a 2 nM final concentration and sequenced on a NextSeq500 instrument (Illumina) using 37 × 37 paired-end mode. After sequencing, the base-called demultiplexed (fastq) read qualities were determined using FastQC (v 0.11.2), aligned to the GENCODE v25 human genome (GRCh38.p7) and gene counts generated using STAR (v 2.5.2a). Post-alignment qualities were generated with QoRTS (v 1.1.6). A expression matrix of raw gene counts was generated using R and filtered to remove low counts genes (defined as those with less than 5 reads in at least one sample). The filtered expression matrix was used to generate a list of differentially expressed genes between the sample groups using DESeq2 package. Reported p values from RNAseq data are from a Wald test adjusted with the Benjamini-Hochberg correction for multiple hypothesis testing. Pathway analysis was performed using DAVID Bioinformatics Resources 6.8.

Quantitative real-time reverse transcription polymerase chain reaction (qRT-PCR) analysis

Total RNA was reverse transcribed into cDNA using the High-Capacity RNA-to-cDNA Kit (Thermo Fisher Scientific). TaqMan Gene Expression Assays for *TNFα* (Hs00174128_m1), *IFNβ* (Hs01077958_s1), *ACTB* (Hs01060665_g1), *Slc12a2* (Mm01265951_m1), *Aqp5*

(Mm00437578_m1) and *Actb* (Mm02619580_g1) were used to calculate the transcript expression. Relative gene expression relative to *ACTB* or *Actb* was calculated using the 2–ΔΔCt method[3]. PCR cycles were performed on the Quantstudio3 Real-Time PCR System (Life Technologies) with the following conditions: 2 minutes at 50°C, 10 minutes at 95°C, 50 cycles of 15 seconds at 95°C and 1 minute at 60°C.

Western blotting

Cultured cells were lysed by incubation for 30 minutes on ice in RIPA buffer with protease and phosphatase inhibitors (Thermo Fisher Scientific), and cleared by centrifugation at 17,000 g for 25 minutes at 4°C. The supernatants were heated for 10 minutes at 97°C in NuPAGE LDS sample buffer, resolved by SDS-PAGE and electrophoretically transferred to polyvinylidene difluoride membranes (Thermo Fisher Scientific). The membranes were blocked with 5% non-fat dried milk for 60 minutes at 25°C, and then incubated at 4°C overnight with primary antibodies: anti-LAMP3 antibody (Proteintech), anti-AQP5 antibody (Alomone Labs), anti-NKCC1 antibody (Cell Signaling Technology) or anti-β-actin antibody (Sigma-Aldrich, USA). After washing three times, the membranes were reacted with rabbit IgG HRP-linked whole antibody (Sigma-Aldrich) for 1 hour at 25°C. Signal was visualized using a Super Signal West Pico Chemiluminescent Substrate (Thermo Fisher Scientific).

Statistical analysis

Quantitative variables were compared by two-tailed *t*-test. Correlation were analyzed using Pearson's correlation coefficient. *P* values less than 0.05 were considered statistically significant. All analyses were performed using the GraphPad PRISM 8.0 software.

REFERENCES

1. Azuma M, Tamatani T, Kasai Y, et al. Immortalization of normal human salivary gland cells with duct-, myoepithelial-, acinar-, or squamous phenotype by transfection with SV40 ori- mutant deoxyribonucleic acid. *Lab Invest* 1993;69(1):24-42.
2. Laine J, Künstle G, Obata T, et al. The protooncogene TCL1 is an Akt kinase coactivator. *Mol Cell* 2000;6(2):395-407.
3. Pfaffl MW. A new mathematical model for relative quantification in real-time RT-PCR. *Nucleic Acids Res* 2001;29(9):e45.

Nakamura H, *et al. Ann Rheum Dis* 2021; 80:1031–1039. doi: 10.1136/annrheumdis-2020-219649

SUPPLEMENTAL FIGURES

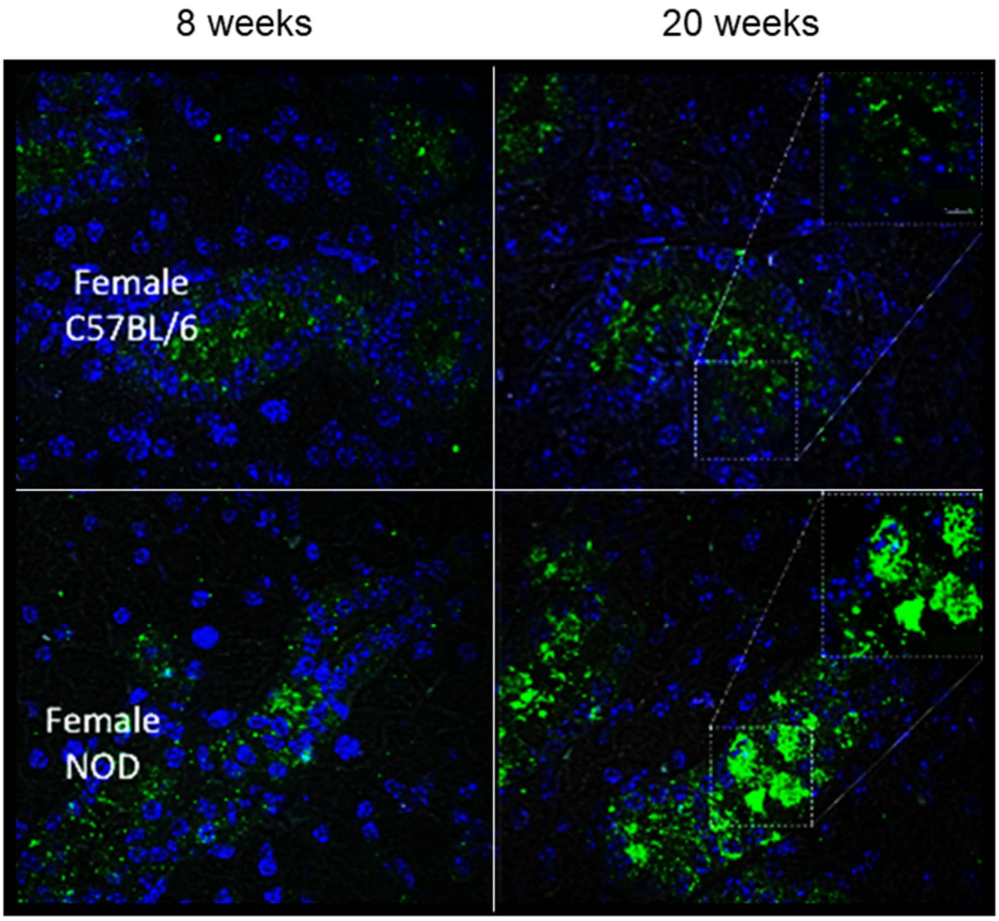

S-Figure 1. Representative immunofluorescence images of LAMP3 (green) and DAPI (blue) in submandibular glands (100× magnification) from non-obese diabetic (NOD) mice and C57BL/6 mice at 8-week or 20-week ages.

Nakamura H, *et al. Ann Rheum Dis* 2021; 80:1031–1039. doi: 10.1136/annrheumdis-2020-219649

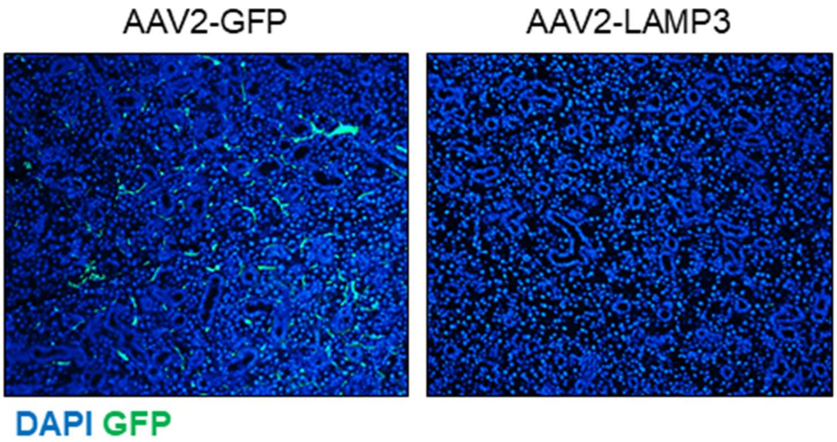

S-Figure 2. Representative immunofluorescence images (10× magnification) of GFP (green) and DAPI (blue) in murine submandibular glands 2 months after transduction with AAV2-GFP or AAV2-LAMP3.

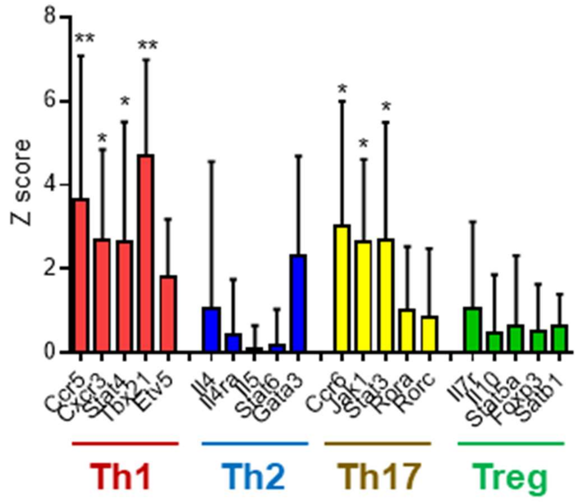

S-Figure 3. Relative gene expression associated with each T-cell subset in submandibular glands from LAMP3 mice 6 months post-cannulation (N = 5) compared with those 2 months post-cannulation (N = 5). Values are shown as the mean ± SD. * $p < 0.05$, ** $p < 0.01$, *t*-test with Bonferroni's correction.

Nakamura H, *et al. Ann Rheum Dis* 2021; 80:1031–1039. doi: 10.1136/annrheumdis-2020-219649

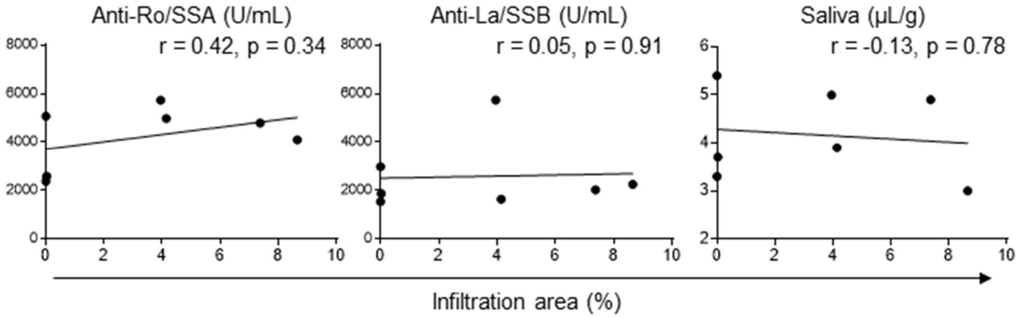

S-Figure 4. Correlation of lymphocytic infiltration area with serum autoantibodies and saliva flow in LAMP3 mice 6 months post-cannulation (N = 7).

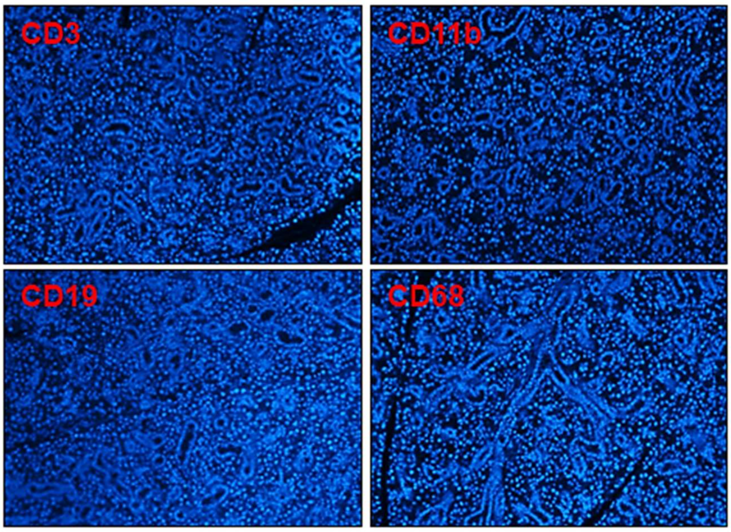

S-Figure 5. Representative immunofluorescence images (20× magnification) in murine submandibular glands 2 months after transduction with AAV2-LAMP3.

Nakamura H, *et al. Ann Rheum Dis* 2021; 80:1031–1039. doi: 10.1136/annrheumdis-2020-219649

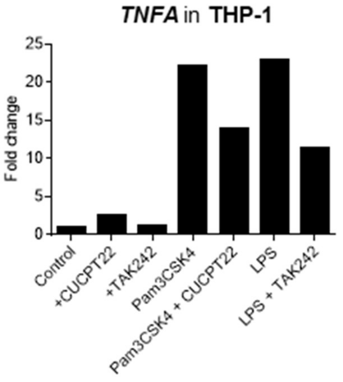

S-Figure 6. TNFα expression in THP-1 stimulated with TLR1/2 agonist (Pam3CSK4, 100 ng/mL) or TLR4 agonist (LPS, 100 ng/mL) ± TLR1/2 antagonist (CUCPT22, 20 μM) or TLR 4 antagonist (TAK242, 40 μM) for 20 hours.

Nakamura H, *et al. Ann Rheum Dis* 2021; 80:1031–1039. doi: 10.1136/annrheumdis-2020-219649

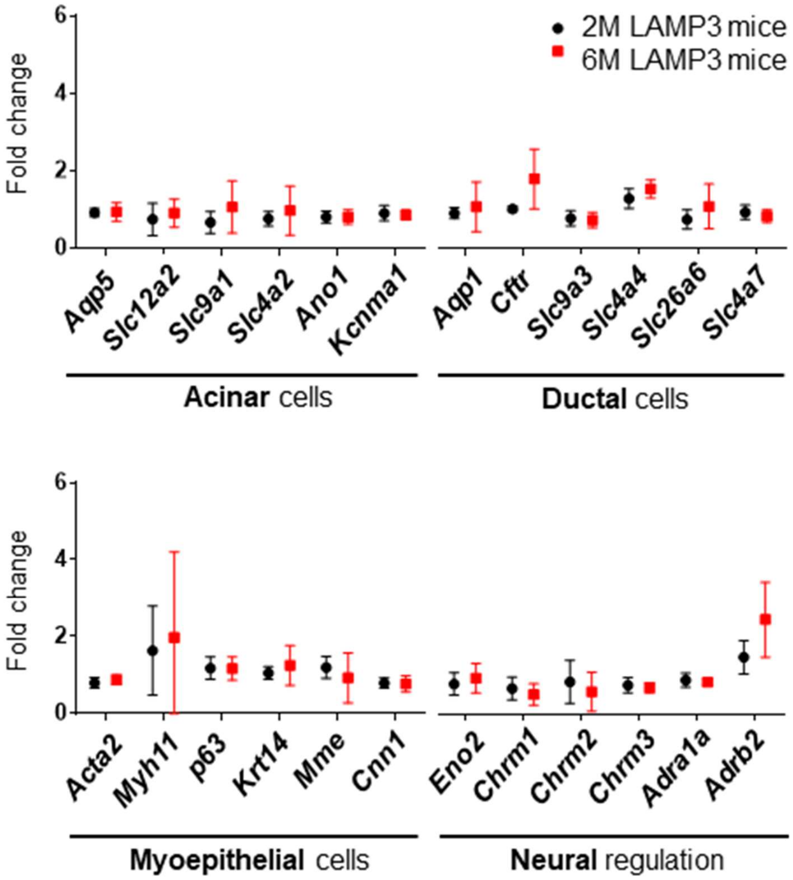

S-Figure 7. Relative gene expression associated with saliva secretion in submandibular glands from LAMP3 mice 2 months (2M) post-cannulation. (N = 5) and 6 months (6M) post-cannulation (N = 5). Values are shown as the mean ± SD, *t*-test with Bonferroni's correction.

Nakamura H, *et al. Ann Rheum Dis* 2021; 80:1031–1039. doi: 10.1136/annrheumdis-2020-219649

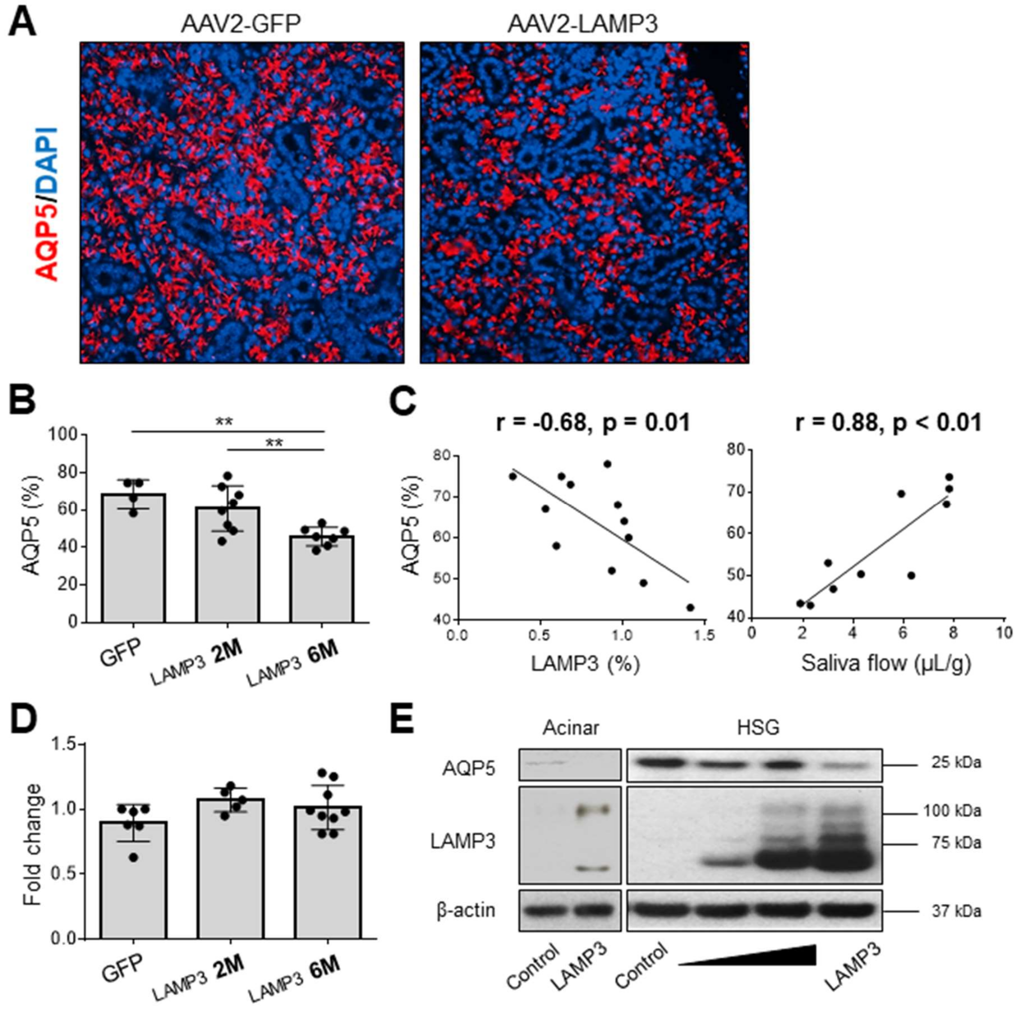

S-Figure 8. LAMP3 expression decreases AQP5 expression in salivary gland epithelial cells. (**A**) Representative immunofluorescence images in murine submandibular glands (40× magnification) from mice treated with AAV2-LAMP3 or AAV2-GFP. (**B**) Expression area of AQP5 was quantified. (**C**) Correlation between the AQP5 and LAMP3 expression in submandibular gland specimens, or saliva flow. (**D**) Transcript change of *Aqp5* gene in submandibular gland tissues. Dots show the result from each murine specimen. Values are shown as the mean ± SD. ** $p < 0.01$, *t*-test with Bonferroni's correction. (**E**) Western blotting analysis of AQP5 and LAMP3 in acinar and HSG cells 72 hours post-transfection with LAMP3 or empty plasmids. Representative data from three independent experiments.

Nakamura H, *et al. Ann Rheum Dis* 2021; 80:1031–1039. doi: 10.1136/annrheumdis-2020-219649
